# Bioinformatics approach and experimental validation reveal the hepatoprotective effect of pachyman against acetaminophen-associated liver injury

**DOI:** 10.18632/aging.205000

**Published:** 2023-09-06

**Authors:** Ka Wu, Jingru Qin, Meizhen Liu, Xin Yan, Chao Guo

**Affiliations:** 1 Department of Pharmacy, The Second People’s Hospital of Nanning City, The Third Affiliated Hospital of Guangxi Medical University, Nanning, China; 2 Department of Clinical Pharmacy, Guigang City People’s Hospital, The Eighth Affiliated Hospital of Guangxi Medical University, Guigang, Guangxi, China; 3 Department of Endocrinology, The Second People’s Hospital of Nanning City, The Third Affiliated Hospital of Guangxi Medical University, Nanning, China

**Keywords:** pachyman, hepatoprotection, acetaminophen, molecular mechanisms, biotargets

## Abstract

Pachyman, known as *Poria cocos* polysaccharides, refers to the bioactive compounds isolated from *Poria cocos*. Pachyman is thought to exert cytoprotective action. However, the detailed mechanisms of pachyman action for hepatoprotection remain unknown. In this study, we aimed to assess the therapeutic actions, molecular mechanisms, and key target proteins of pachyman in the treatment of liver injury through network pharmacology and molecular docking assays. Furthermore, these bioinformatic findings were validated by an acetaminophen (APAP)-induced liver injury *in vivo*. Primarily using bioinformatic analysis, we screened and characterized 12 genes that act as potential therapeutic targets of pachyman against APAP-induced liver injury, in which all core targets were obtained. By using enrichment analysis, these core target genes of pachyman were characterized to reveal the pharmacological functions and molecular mechanisms of anti-liver injury induced by APAP. A molecular docking simulation was further performed to certain anti-liver injury target proteins of pachyman, including cytochrome P450 3A4 enzyme (*CYP3A4*) and inducible nitric oxide synthase *(NOS2)*. In animal experiments, pachyman exerted potent hepatoprotective activities in prenatal APAP-exposed offspring livers, characterized by activated hepatocellular *CYP3A4* and *NOS2* expressions. These current findings have thus indicated that pachyman exerts hepatoprotective effects and may be the promising nutraceuticals for the treatment of APAP-induced liver injury.

## INTRODUCTION

In the body, the liver tissue exhibits potent detoxification and medicant metabolism, and thus hepatocytes may be sensitized to certain drug metabolites, resulting in potential drug-induced liver injury [1]. APAP, chemically termed as N-acetyl-p-aminophenol, is a non-prescribed drug for antipyretic and analgesic applications. It can cause dose-dependent toxic effects in unconscionable use [2]. Statistically, APAP-associated hepatotoxicity accounts for most acute liver failure cases in the developed western countries [3]. Owing to potential intoxication of APAP, the self-directed use of APAP for children should be great concern as the toxic metabolite of N-acetyl-p-benzo-quinone imine (NAPQI) can be released [4]. APAP is typically safe within the recommended dosage as it is metabolized by the hepatocytes before physiological excretion [5]. However, overdosage use of APAP may trigger potential hepatotoxicity via inducing underlying pathological events including oxidative stress, microsomal dysfunction, and inflammatory impairment [6]. In clinical application, N-acetylcysteine (NAC, a strong antioxidant) is prescribed for treating APAP-caused hepatotoxicity. In addition, therapeutic effectiveness of NAC may be achieved through enhancing hepatocellular glutathione content and reducing NAPQI expression [7]. NAC can effectively treat acute overdose of APAP; however, APAP-induced hepatotoxicity may be chronic and lead to liver failure over time. Moreover, intravenous injection of NAC can cause the appearance of urticaria, itching, or other side-effects [8]. Thus, there is an urgent need to explore and identify the bioactive ingredient sourced from natural products that is use for managing APAP-triggered liver injury. *Poria cocos*, known as “Fuling” in Chinese, is a nutraceutical supplement that has been used as a Chinese folk medicine [9]. *Poria cocos* is commonly used to manage some hepatobiliary disorders, such as hepatitis and cholestasis [10]. Pachyman, an extract of *Poria cocos* plant, comprises functional polysaccharides characterized with beneficial effects, including anti-cancer activity [11], immunostimulatory action [12], and positive metabolic modulation [13]. A previous report showed that pachyman suppresses alcoholic liver impairment *in vivo* by regulating cytochrome P450 2 E1 (CYP2 E1) expression and inactivating the NF-κB inflammatory pathway [14]. Our early findings suggest that pachyman, a group of *Poria cocos* polysaccharides, achieved antihepatotoxic benefits against APAP-lesioned liver cells *in vivo* and *in vitro*. More interestingly, the underlying anti-liver injury mechanisms are found with the involvement of inhibiting cell death, suppressing hepatic inflammatory reaction, and modulating heat shock protein 90 activity [15, 16]. However, more detailed mechanisms regarding pachyman against APAP-associated liver injury remain unrevealed utterly. Therefore, it is of great necessity to define the new pharmacological targets of pachyman against APAP-induced liver injury. Frontier bioinformatics analyses including network pharmacology and molecular docking have highlighted the systematic strategies to detail multiple targets and molecular mechanisms of bioactive agents in the treatment of clinical disorders [17]. Based on network pharmacology-obtained findings, the therapeutic biotargets of natural candidate products against diseases have been revealed, including those of calycosin against cerebral ischemia/reperfusion injury [18], calycosin against osteosarcoma [19], and licorice against COVID-19 [20]. More attentively, our previous report by using network pharmacology approach has been revealed the pharmacological targets and mechanisms of curcumol for treating interstitial cystitis [21]. In this study, network pharmacology and molecular docking analyses were performed for the stepwise revelation of key targets and molecular mechanisms of pachyman against APAP-induced liver injury in detail. Furthermore, APAP-exposed livers in pregnant mice were used to identify the pharmacological actions and biotargets of pachyman.

## MATERIALS AND METHODS

### Drug (pachyman) target gene collection

The three available databases including Swiss Target Prediction (http://www.swisstargetprediction.ch) and, SuperPred (https://prediction.charite.de/index.php) PharmMapper were employed to screen out the putative genes of pachyman. These putative targets were reviewed in the Swiss-Prot/UniprotKB database [22], and the repetition genes were removed by exclusion setting.

### Disease (APAP-induced liver injury) target gene selection

Following an earlier study, the keyword of “APAP-induced liver injury” was imported to Online Mendelian Inheritance in Man (OMIM, http://omim.org/), GeneCards (http://www.genecards.org/) and Pharmacogenomics Knowledgebase (PharmGKB, http://www.pharmgkb.org/) databases to obtain liver injury-associated target genes. Finally, Venn mapping analysis [23] was performed between the target genes of pachyman and APAP-induced liver injury to identify the common genes. Species was limited to “Homo sapiens”.

### Gene ontology (GO) function and kyoto encyclopedia of genes and genomes (KEGG) pathway analyses

Functional annotation, enrichment analysis, and visualization in these target genes were achieved using the “ClusterProfiler”, “org.Hs.eg.Db”, and “ggplot2” tools in R-language packages [24]. Enrichment data with *p*-value of less than 0.05 was used for figure plotting.

### Core target gene identification and network construction

All core genes associated with pachyman against APAP-induced liver injury were identified using the Network Analyzer plug-in of Cytoscape software (3.8.2 version) and previous procedures [25]. Another protein-protein interaction (PPI) network was constructed via the String database for visualization of analytical data [26].

### Molecular docking determination

The chemical structure of pachyman was harvested by using the PubChem database (https://pubchem.ncbi.nlm.nih.gov). In this design, the top 3 core target proteins were selected for molecular docking verification, and the corresponding protein structures were obtained from the Protein Data Bank database (http://www.rcsb.org/pdb/). The force field was optimized using the three-dimensional structure of the compound downloaded by the ChemBio3D Draw module in the Chem Bio Office2010 software [27]. The AutoDockTools (1.5.6 version) in AutoDock Vina software [28] was used to process the core target proteins for hydrogenation, and Gasteiger charge before merging with non-polar hydrogens. According to the root mean square deviation (RMSD) of the docked ligand molecule and the original ligand molecule, the rationality of the docking parameter settings was judged reasonably.

### Animal study and treatment

After seven days of adaptive acclimatization for adult C57BL/6 mice (Hunan STA Lab Animal Company, China), male and female mice (1:1) were caged for breeding, and then the vaginal plug status of female mice was checked in the next morning. The female mice with obvious vaginal plugs were selected as pregnancy [29] before being kept in clean cages alone (the pregnancy time was calculated as E0.5 at this time). At E11.5, the offspring mice were randomly divided into different groups (*n* = 5). And a prenatal APAP exposure group were given 300 mg/kg concentration of APAP dissolved in sterile physiological saline solution every week for 3 weeks; The pachyman (Yuanye, Shanghai, China; dissolved in sterile physiological saline solution) treatment group in prenatal APAP exposure mice was given 400 mg/kg by gavage every day for 21 days (the entire pregnancy). The dose of APAP used in this experiment was lower than the clinically recommended or commonly used dose. And the treatment dose of pachyman was referenced as our previous study [15]. Fetal mouse liver tissue was collected at E12.5, and E18.5 time periods (critical period of liver development) during pregnancy, and the liver sections were tested with immunostaining examination. Other pregnant mice were reared until parturition, and liver tissues from the neonatal mice (PND1; postnatal day 1) were harvested and prepared as sections for subsequent immunofluorescence assays.

### Immunostaining procedure

In brief, the liver specimens from E12.5, E18.5 and PND1 were isolated for preparing paraffin block before performing microtome section. Subsequently, 5 μm paraffin-embedded sections were subjected to deparaffinization and gradient dehydration. After being blocked with fresh bovine serum albumin buffer (5%, v/v), the dewaxed sections were hatched with primary antibodies against Cyp4a, p-AKT, mTOR, IRβ, IGF-1R, FGF21, CYP3A4, and NOS2 (1:150–200 dilutions; Bioss, Beijing, China) at 4°C overnight. After being washed with neutral phosphate buffered saline buffer, the sections were re-hatched with secondary antibody-coupled fluorescent dye (Beyotime Biotechnology, China) under light-free conditions for approximately 1 h at indoor temperature. Subsequently, 4′,6-diamidino-2-phenylindole (DAPI, Beyotime, China) dye was used for nuclear staining, in which the positive cells were identified under different magnification view and counted for statistic determination, as previously reported [30].

### Statistical analysis

All final data were denoted as the mean ± standard deviation (SD) and were processed by using *Statistical Product Service Solutions* 21.0 (Chicago, IL, USA). One-way analysis of variance (ANOVA) followed by least significant difference (LSD) *post hoc* test was employed to compare the statistical difference within at least two groups, characterized with statistical significance when a *p*-value less than 0.05.

## RESULTS

### Pachyman-liver injury target identification data

**Figure 1:**
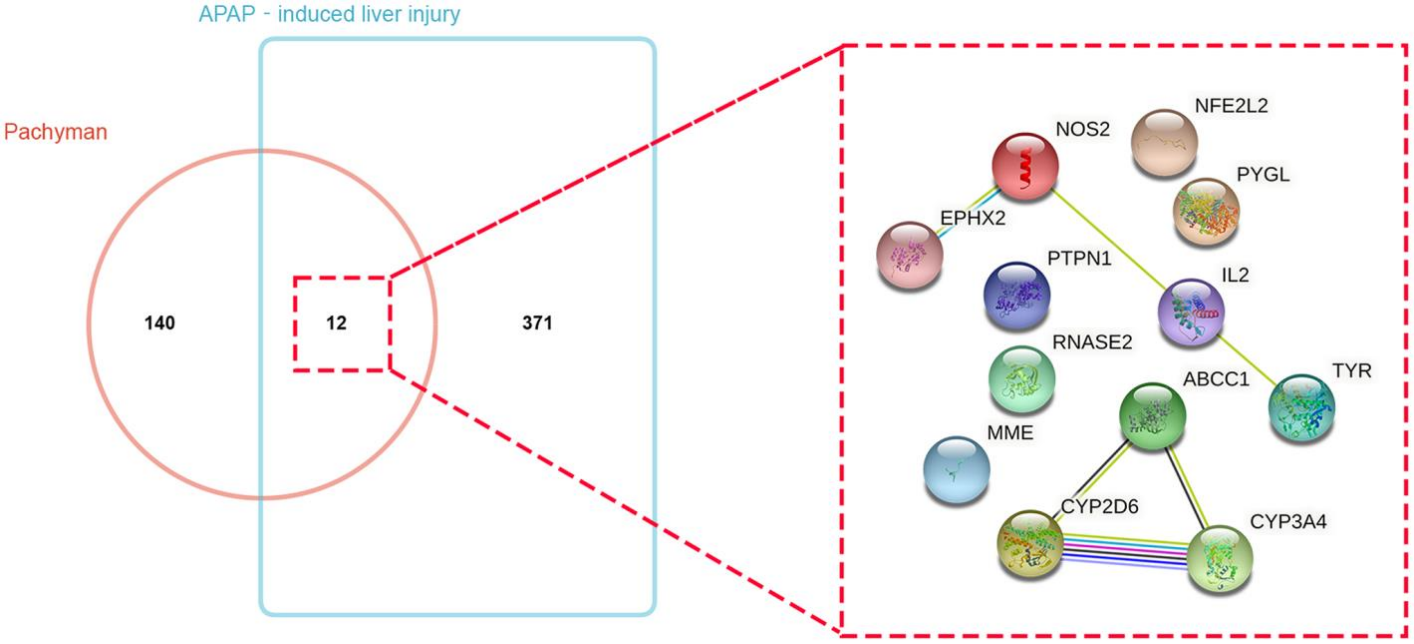
Bioinformatics and Venn mapping analyses showed 152 and 383 respective genes in pachyman and APAP-induced liver injury, and 12 intersection genes were identified and gene interaction network diagram of 12 potential targets was visualized.

The putative target genes in pachyman were collected from the respective databases, and 152 genes of pachyman were identified. Meanwhile, 383 APAP-induced liver injury genes were obtained. By assaying the intersection of the drug and disease targets, a total of 12 common or overlapping genes were acquired from the Venn mapping analysis: CYP3A4, ABCC1, NOS2, NFE2L2, TYR, EPHX2, RNASE2, PYGL, IL2, MME, PTPN1, and CYP2D6, as shown in Figure 1.

### Enrichment analysis reports

**Figure 2:**
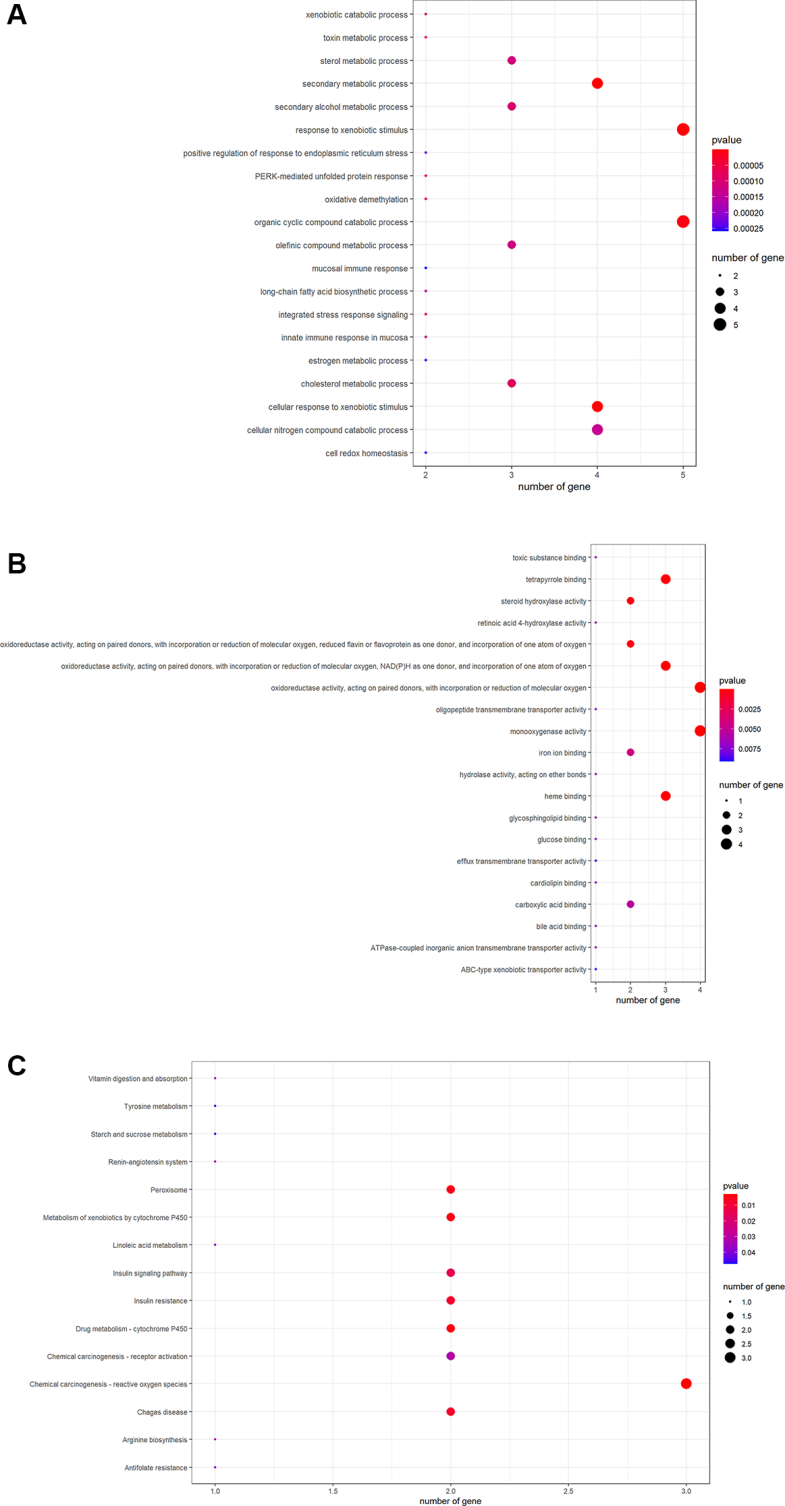
The data from GO functional analysis were sorted in ascending sequence of *p*-value, and the top annotations were highlighted accordingly in bubble diagrams, as showed in biological process annotations (**A**), cellular component annotations (**B**), molecular function annotations (**C**).

**Figure 3:**
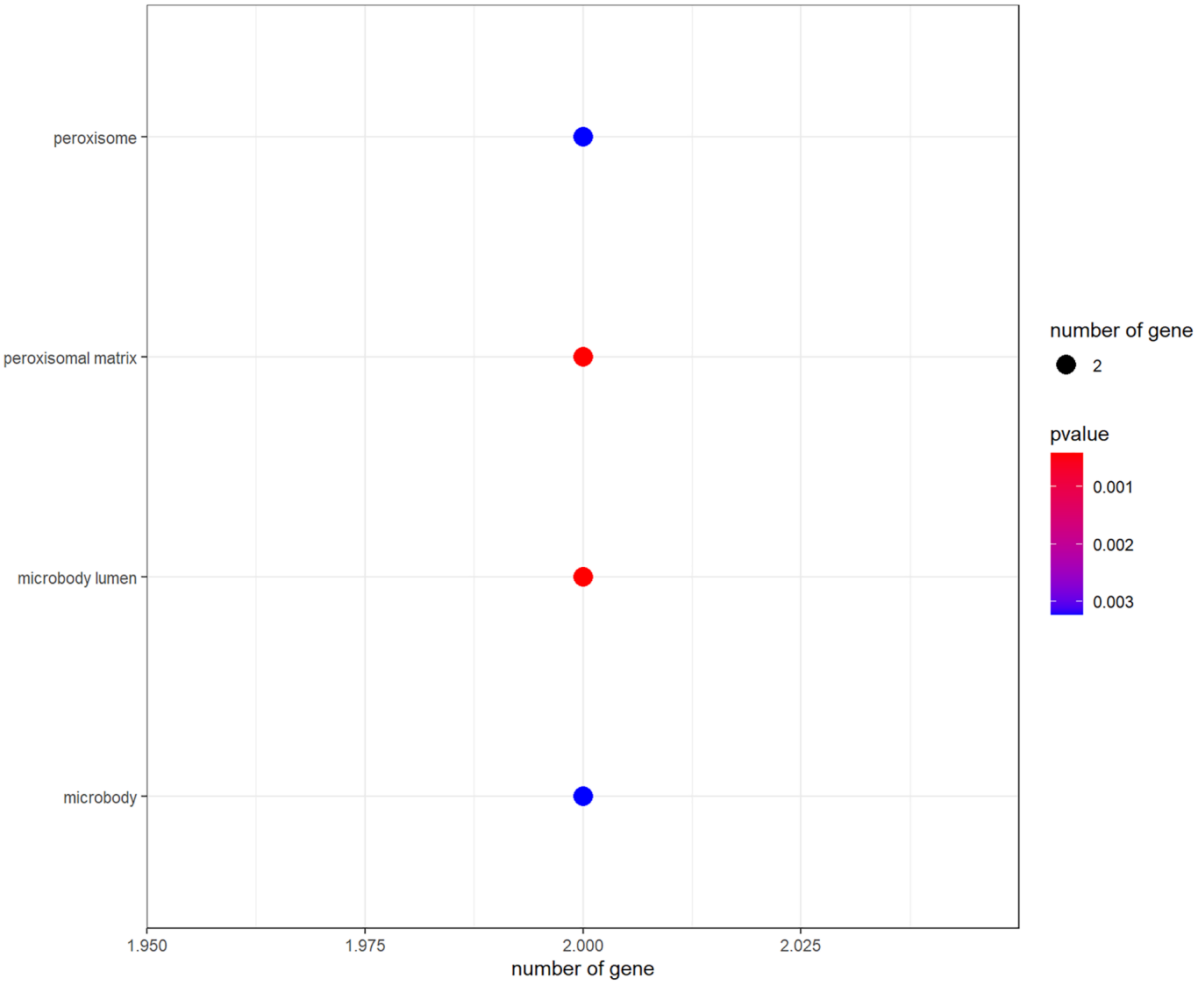
All main signaling pathways were exhibited through KEGG pathway analysis. After ordering based on the *p*-value, the top molecular pathways were presented for mapping.

R-language software was used to conduct GO and KEGG assays to disclose the beneficial functions and mechanisms of pachyman against APAP-induced liver injury. All top biological processes (BPs), cellular components (CCs), and molecular functions (MFs) in GO annotations were illustrated in Figure 2A–2C, respectively. Other main signaling pathways in KEGG enrichment were presented in Figure 3. As per KEGG findings (*p*-value < 0.05), the pachyman-exerted anti-liver injury action against APAP might mainly involve chemical carcinogenesis-reactive oxygen species, drug metabolism-cytochrome P450, metabolism of xenobiotics by cytochrome P450, peroxisome, insulin resistance, insulin signaling pathway, arginine biosynthesis, renin-angiotensin system, chemical carcinogenesis-receptor activation, vitamin digestion and absorption, linoleic acid metabolism, antifolate resistance, tyrosine metabolism, and starch and sucrose metabolism. In addition, BPs were largely related to secondary metabolic process, cellular response to xenobiotic stimulus, organic cyclic compound catabolic process, toxin metabolic process, PERK-mediated protein response, oxidative demethylation, cholesterol metabolic process, integrated stress response signaling, and secondary alcohol metabolic process. CCs were mostly involved in peroxisomal matrix, microbody lumen, peroxisome, microbody. MFs were chiefly implicated in monooxygenase activity, oxidoreductase activity, acting on paired donors, with incorporation or reduction of molecular oxygen, NAD(P)H as one donor, and incorporation of one atom of oxygen, heme binding, tetrapyrrole binding, steroid hydroxylase activity, reduced flavin or flavoprotein as one donor, and incorporation of one atom of oxygen, iron ion binding, carboxylic acid binding, and retinoic acid 4-hydroxylase activity.

### Core targets’ identification

**Figure 4:**
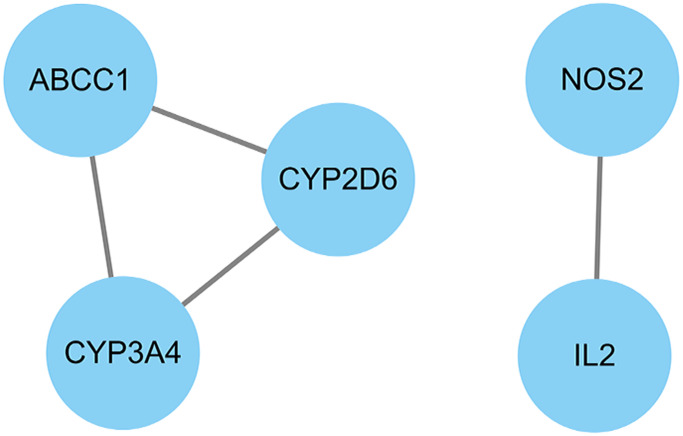
All core target genes from pachyman against APAP-induced liver injury were showed in connection diagram.

We used Cytoscape software to calculate the topological parameters of pachyman against APAP-induced liver injury targets and function-related protein interaction network. The median degree of freedom of these targets was 2, and the maximum degree of freedom was 2. Based on parametrical analysis, all 5 core targets were finally obtained, including ABCC1, CYP3A4, CYP2D6, NOS2, and IL2 (Figure 4).

### Molecular docking results

**Figure 5:**
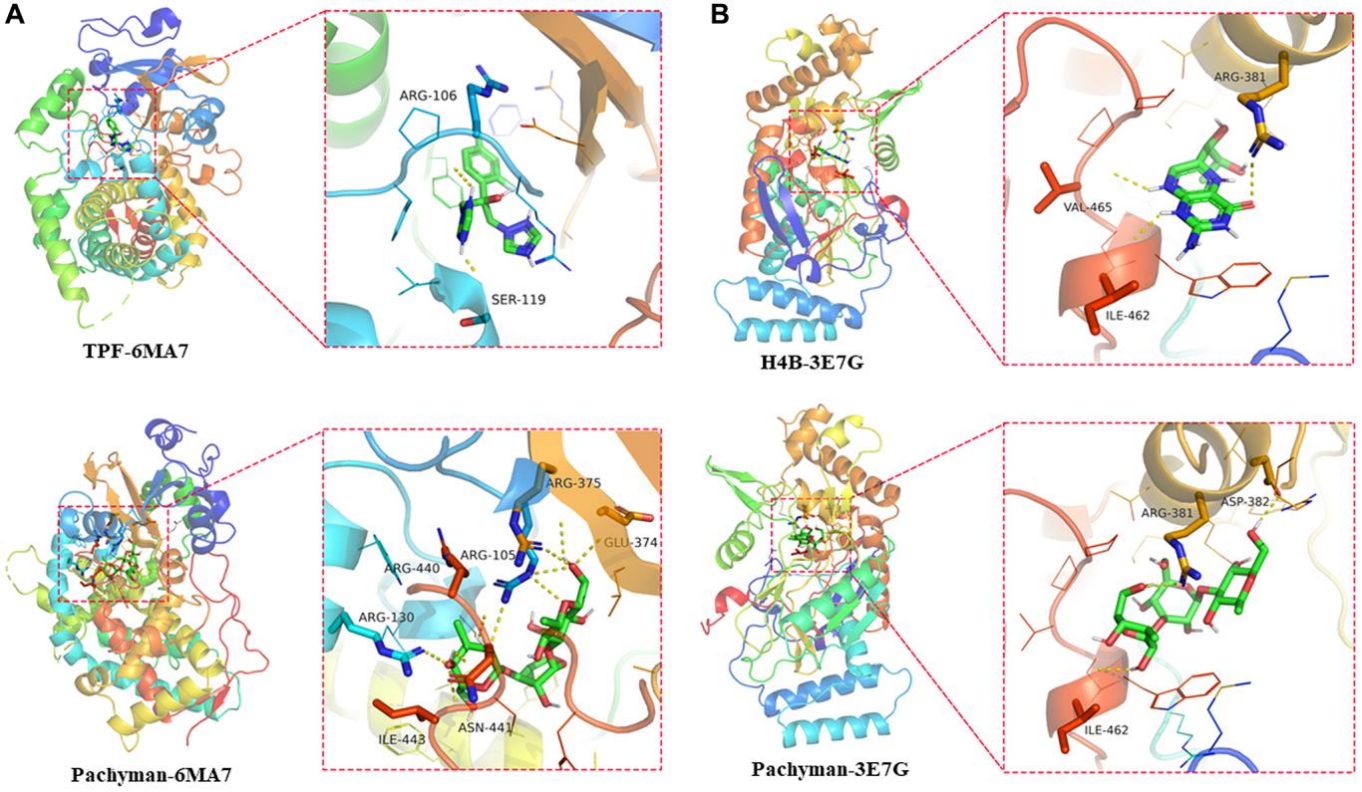
Docking features in pachyman and core target proteins, and interaction between pachyman and target proteins of CYP3A4 and NOS2, accompanied with the binding site of 6MA7 (**A**) and 3E7G (**B**).

The interaction and binding models of core target proteins and pachyman can be characterized at the molecular and spatial levels. We discovered the findings including CYP3A4 (TPF-6MAT: ARG-106 (2.7 Å), SER-119 (2.1 Å), free docking score: −6.5 Kcal/mol; Pachyman-6MAT: GLU-374 (2.8 Å), ARG-375 (3.2 Å), ARG-105 (3.1 Å), ARG-440 (2.8 Å), ASN-441 (2.6 Å), ARG -130 (2.8 Å), ILE-443 (3.0 Å), free docking score: −7.4 Kcal/mol) (Figure 5A) and NOS2 (H4B-3E7G: RG-381 (2.8 Å), VAL-465 (2.4 Å), ILE-462 (2.2 Å), free docking score: −5.6 Kcal/mol; Pachyman-3E7G: ARG-381 (2.8 Å), ASP-382 (2.1 Å), ILE-462 (2.3 Å), free docking score: −6.5 Kcal/mol) (Figure 5B) resulted in potent binding sites in the target proteins. These *in-silico* data suggested that pachyman could bind tightly to CYP3A4 and NOS2.

### Pharmacological activities of pachyman against APAP-related liver injury

**Figure 6:**
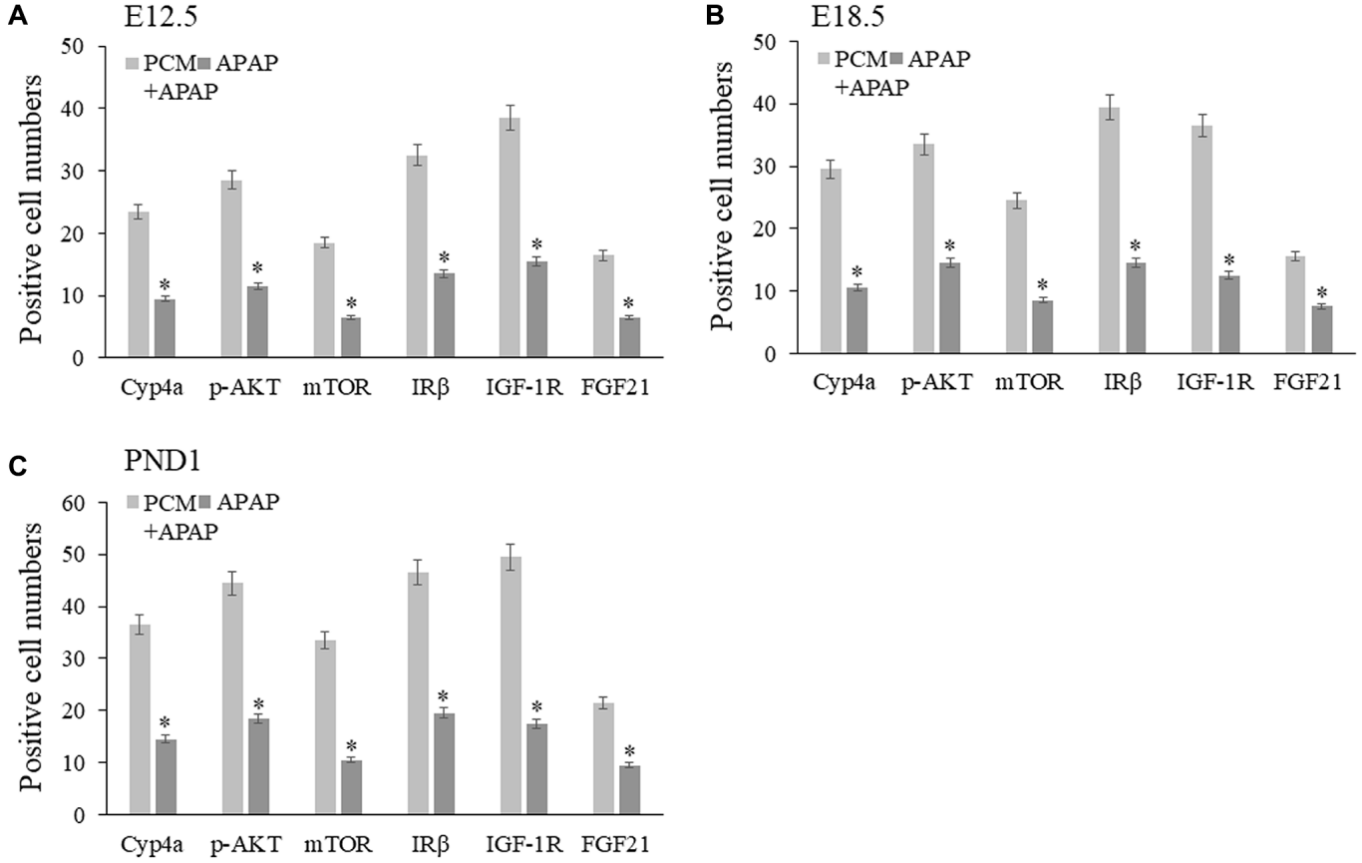
Pachyman-treated APAP-exposed mice *in utero* showed increased Cyp4a, p-AKT, mTOR, IRβ, IGF-1R, FGF21 positive protein expressions in E12.5 livers (**A**), E18.5 livers (**B**), and PND1 livers (**C**). Abbreviation: PCM: pachyman treatment.

**Figure 7:**
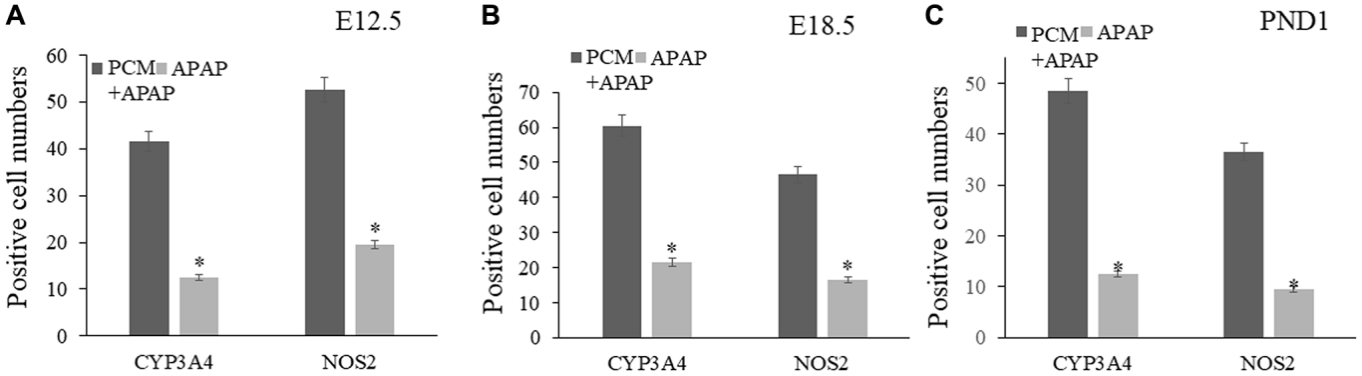
Pachyman-treated livers from prenatal APAP exposure resulted in elevated positive expressions of CYP3A4, and NOS2 in E12.5 livers (**A**), E18.5 livers (**B**), and PND1 livers (**C**). Abbreviation: PCM: pachyman treatment.

We established a prenatal APAP exposure model to validate the pharmacological activity and hepatoprotective action of pachyman. It was observed that the pachyman-treated APAP-exposed mice showed increased positive protein expressions of Cyp4a, p-AKT, mTOR, IRβ, IGF-1R, and FGF21 in the offspring livers (E12.5, E18.5, and PND1) when compared with those expressions in APAP-exposed controls (*P* < 0.05) (Figure 6). To further validate the *in-silico* docking findings, reduced intrahepatic positive protein expressions of CYP3A4 and NOS2 were detected in prenatal APAP-exposed offspring mice (*P* < 0.05), and pachyman-treated livers (E12.5, E18.5, and PND1) showed elevated positive protein expressions of CYP3A4 and NOS2, when compared to the controls (*P* < 0.05) (Figure 7).

## DISCUSSION

Drug-induced liver injury (DILI), such as APAP-induced by DILI, is one of the leading causes of acute liver injury induced by hepatotoxicity in long-time or high-dose use of medicine [31]. Statistical data show that the incidence of DILI in mainland China is elevated in recent years and the reported cases are greater than those in Western countries [32]. Some clinical medications such as ursodeoxycholic acid, silymarin and glycyrrhizin are used previously for DILI treatment, and the treatment for acute DILI can be prescribed with NAC [33]. DILI-associated pathological outcomes may include inflammatory infiltration, oxidative stress, hepatic apoptosis, and necrosis, eventually resulting in liver dysfunction or liver failure [34]. APAP, a classical antipyretic/analgesic medicine, can trigger life-threatening liver injury when overdosed or long-term use [35]. Clinical antidote NAC is prescribed for the therapy of DILI, including APAP-caused cytotoxicity [36]. However, NAC administration may affect the overall therapeutic effect when DILI worsens or becomes chronic. Thus, some of potential agents should be further explored and identified for management of DILI. In China, pachyman, the bioactive ingredient of *Poria cocos*, has been used for hepatoprotection based on the beneficial mechanisms of anti-oxidative and anti-inflammatory actions [37]. However, more in-depth anti-liver injury mechanisms of pachyman remain unrevealed. By screening out all core targets for GO-functional and KEGG-enriched analyses, we identified that the hepatoprotective mechanism of pachyman might be associated with the regulation of metabolism of xenobiotics by cytochrome P450, insulin resistance, and the insulin signaling pathway. In APAP-induced liver injury model of mice offspring, we found that pachyman-treated prenatal APAP-exposed offspring mice (E11.5, E18.5, PND1) showed increased Cyp4a, p-AKT, mTOR, IRβ, IGF-1R, and FGF21 protein expression in the liver sections. These altered molecular protein expressions suggested that pachyman exerted direct benefits against APAP-associated liver impairment via improving the functions of metabolism and molecular hormone. It is found that supplementary FGF21 may protect against iron overload-caused mitochondria dysfunction in liver cells, and liver damage through suppression of ferroptosis [38]. Additionally, FGF21 may reduce the acute liver injury (ALI) via activating the sirtuin type 1 (SIRT1)-autophagy molecular pathway [39]. Current biochemical data suggested that the hepatoprotective action of pachyman might be attributed to the enhanced detoxification ability and metabolic efficacy, in which these findings were consistent with the results mentioned above. Bioinformatic analysis using network pharmacology and molecular docking approach was applied to identify the key pharmacological proteins (targets) associated with pachyman against APAP-induced liver impairment. These core pharmacological proteins included *CYP3A4*, and *NOS2*. CYP3A4, mainly occurs in the liver and gut, is involved in the metabolism of drugs [40]. It is reported that hepatic drug-metabolizing enzymes, such as CYP3A4, can catalyze the biotransformation of APAP for biological detoxification [41]. The marked suppression of CYP3A2 mRNA expression can be achieved in the extracts of *Poria cocos* [42]. NOS2, a nitric oxide synthase expressed in the liver, may assist macrophages in the immune system to fight against pathogens via regulation of oxidative stress of nitric oxide (free radicals) [43]. In addition, NOS2 may be used for suppressing APAP-induced hepatotoxicity through reducing the inflammation in activated neutrophils [44]. Additionally, NOS2 can be activated in mouse macrophages following *Poria cocos* polysaccharide [45]. Therefore, we speculate that CYP3A4 and NOS2 may be the pharmacological targets of pachyman against APAP-induced liver injury based on molecular docking analysis and reference report. Our *in vivo* data suggested that prenatal treatment of pachyman elevated hepatocellular CYP3A4 and NOS2 expressed activities in the mice offspring exposed to APAP *in utero*. Prenatal drug exposure is an effective research model for functional assessment in organs [46]. These experimental data suggest that detoxification and anti-inflammatory action were achieved by pachyman against APAP-associated liver injury. Collectively, these preclinical data indicate that pachyman strengthens the detoxification function and immunoregulation activity via activating key protein expressions of CYP3A4 and NOS2 in liver tissue. Thus, it may be within reason to conclude that the anti-liver injury action of pachyman against APAP-induced hepatotoxicity is associated with the enhancement of hepatocellular detoxification and immunologic function. However, several other *in vitro* and *in vivo* experiments also need to be designed and conducted to validate the current bioinformatic findings before clinical evaluation for pachyman use.

## CONCLUSION

In the current report, the pharmacological mechanisms of pachyman were revealed through bioinformatic analysis and then partly verified by inducing liver injury with APAP in offspring mice from pregnant models. We detected experimentally that pachyman might meliorate the detoxifying function via activating Cyp4a, CYP3A4, metabolic function via activating AKT, mTOR, IRβ, IGF-1R, FGF21, and immunity function via activating NOS2. Therefore, we conclude that pachyman, the bioactive ingredients of *Poria cocos*, may be effectively used for the potential treatment of APAP-induced liver injury.

## References

[1] Tillmann HL, Rockey DC. Signatures in drug-induced liver injury. Curr Opin Gastroenterol. 2020; 36:199–205. 10.1097/MOG.0000000000000636 32205565 PMC10896173

[2] Bunchorntavakul C, Reddy KR. Acetaminophen (APAP or N-Acetyl-p-Aminophenol) and Acute Liver Failure. Clin Liver Dis. 2018; 22:325–46. 10.1016/j.cld.2018.01.007 29605069

[3] Jaeschke H. Acetaminophen: Dose-Dependent Drug Hepatotoxicity and Acute Liver Failure in Patients. Dig Dis. 2015; 33:464–71. 10.1159/000374090 26159260 PMC4520394

[4] Marzuillo P, Guarino S, Barbi E. Paracetamol: a focus for the general pediatrician. Eur J Pediatr. 2014; 173:415–25. 10.1007/s00431-013-2239-5 24374658

[5] Wang X, Wu Q, Liu A, Anadón A, Rodríguez JL, Martínez-Larrañaga MR, Yuan Z, Martínez MA. Paracetamol: overdose-induced oxidative stress toxicity, metabolism, and protective effects of various compounds in vivo and in vitro. Drug Metab Rev. 2017; 49:395–437. 10.1080/03602532.2017.1354014 28766385

[6] Ramachandran A, Jaeschke H. Acetaminophen Hepatotoxicity. Semin Liver Dis. 2019; 39:221–34. 10.1055/s-0039-1679919 30849782 PMC6800176

[7] Codinach-Martín M, Ortega-Pérez J, Gispert-Ametller MÀ, Salgado-García E, Rodríguez-Mariblanca A, Nogué-Xarau S, Puiguriguer-Ferrando J. N-acetylcysteine as an antidote for paracetamol poisoning: a multicenter study. Emergencias. 2022; 34:190–5. 35736523

[8] Johnson MT, McCammon CA, Mullins ME, Halcomb SE. Evaluation of a simplified N-acetylcysteine dosing regimen for the treatment of acetaminophen toxicity. Ann Pharmacother. 2011; 45:713–20. 10.1345/aph.1P613 21586653

[9] Li L, Zuo ZT, Wang YZ. The Traditional Usages, Chemical Components and Pharmacological Activities of *Wolfiporia cocos*: A Review. Am J Chin Med. 2022; 50:389–440. 10.1142/S0192415X22500161 35300566

[10] Wei C, Qiu J, Wu Y, Chen Z, Yu Z, Huang Z, Yang K, Hu H, Liu F. Promising traditional Chinese medicine for the treatment of cholestatic liver disease process (cholestasis, hepatitis, liver fibrosis, liver cirrhosis). J Ethnopharmacol. 2022; 297:115550. 10.1016/j.jep.2022.115550 35863612

[11] Qin L, Huang D, Huang J, Qin F, Huang H. Integrated Analysis and Finding Reveal Anti-Liver Cancer Targets and Mechanisms of Pachyman (*Poria cocos* Polysaccharides). Front Pharmacol. 2021; 12:742349. 10.3389/fphar.2021.742349 34603055 PMC8484528

[12] Liang J, Zhao M, Xie S, Peng D, An M, Chen Y, Li P, Du B. Effect of steam explosion pretreatment on polysaccharide isolated from Poria cocos: Structure and immunostimulatory activity. J Food Biochem. 2022; 46:e14355. 10.1111/jfbc.14355 35892192

[13] Wang J, Zheng D, Huang F, Zhao A, Kuang J, Ren Z, Chen T, Lei J, Lin J, Wang X, Jia W, Xie G, Zheng X. Theabrownin and *Poria* cocos Polysaccharide Improve Lipid Metabolism *via* Modulation of Bile Acid and Fatty Acid Metabolism. Front Pharmacol. 2022; 13:875549. 10.3389/fphar.2022.875549 35833020 PMC9271858

[14] Jiang YH, Zhang Y, Wang YY, Zhang WX, Wang MW, Liu CQ, Peng DY, Yu NJ, Wang L, Chen WD. [Extracts of Poria cocos polysaccharides improves alcoholic liver disease in mice via CYP2E1 and NF-κB inflammatory pathways]. Zhongguo Zhong Yao Za Zhi. 2022; 47:134–40. 10.19540/j.cnki.cjcmm.20210930.402 35178920

[15] Wu K, Fan J, Huang X, Wu X, Guo C. Hepatoprotective effects exerted by Poria Cocos polysaccharides against acetaminophen-induced liver injury in mice. Int J Biol Macromol. 2018; 114:137–42. 10.1016/j.ijbiomac.2018.03.107 29572139

[16] Wu K, Guo C, Yang B, Wu X, Wang W. Antihepatotoxic benefits of Poria cocos polysaccharides on acetaminophen-lesioned livers in vivo and in vitro. J Cell Biochem. 2019; 120:7482–8. 10.1002/jcb.28022 30378160

[17] Zhao L, Zhang H, Li N, Chen J, Xu H, Wang Y, Liang Q. Network pharmacology, a promising approach to reveal the pharmacology mechanism of Chinese medicine formula. J Ethnopharmacol. 2023; 309:116306. 10.1016/j.jep.2023.116306 36858276

[18] Yu S, Wu K, Liang Y, Zhang H, Guo C, Yang B. Therapeutic targets and molecular mechanism of calycosin for the treatment of cerebral ischemia/reperfusion injury. Aging (Albany NY). 2021; 13:16804–15. 10.18632/aging.203219 34176787 PMC8266369

[19] Pan Q, Wu K, Tan J, Li Y, Liang X, Su M. Anti-neoplastic characteristics and potential targets of calycosin against bisphenol A-related osteosarcoma: bioinformatics analysis. Bioengineered. 2021; 12:4278–88. 10.1080/21655979.2021.1956401 34311656 PMC8806932

[20] Fu Y, Fang Y, Gong S, Xue T, Wang P, She L, Huang J. Deep learning-based network pharmacology for exploring the mechanism of licorice for the treatment of COVID-19. Sci Rep. 2023; 13:5844. 10.1038/s41598-023-31380-7 37037848 PMC10086012

[21] Wu K, Wei P, Liu M, Liang X, Su M. To reveal pharmacological targets and molecular mechanisms of curcumol against interstitial cystitis. J Adv Res. 2019; 20:43–50. 10.1016/j.jare.2019.05.003 31193808 PMC6543129

[22] Boutet E, Lieberherr D, Tognolli M, Schneider M, Bairoch A. UniProtKB/Swiss-Prot. Methods Mol Biol. 2007; 406:89–112. 10.1007/978-1-59745-535-0_4 18287689

[23] Pelikan A, Herzel H, Kramer A, Ananthasubramaniam B. Venn diagram analysis overestimates the extent of circadian rhythm reprogramming. FEBS J. 2022; 289:6605–21. 10.1111/febs.16095 34189845

[24] Giorgi FM, Ceraolo C, Mercatelli D. The R Language: An Engine for Bioinformatics and Data Science. Life (Basel). 2022; 12:648. 10.3390/life12050648 35629316 PMC9148156

[25] Li C, Wang C, Guo Y, Wen R, Yan L, Zhang F, Gong Q, Yu H. Research on the effect and underlying molecular mechanism of Cangzhu in the treatment of gouty arthritis. Eur J Pharmacol. 2022; 927:175044. 10.1016/j.ejphar.2022.175044 35643303

[26] Wang T, Yang N, Liang C, Xu H, An Y, Xiao S, Zheng M, Liu L, Wang G, Nie L. Detecting Protein-Protein Interaction Based on Protein Fragment Complementation Assay. Curr Protein Pept Sci. 2020; 21:598–610. 10.2174/1389203721666200213102829 32053071

[27] Kerwin SM. ChemBioOffice Ultra 2010 suite. J Am Chem Soc. 2010; 132:2466–7. 10.1021/ja1005306 20121088

[28] Oo A, Hassandarvish P, Chin SP, Lee VS, Abu Bakar S, Zandi K. In silico study on anti-Chikungunya virus activity of hesperetin. PeerJ. 2016; 4:e2602. 10.7717/peerj.2602 27812412 PMC5088613

[29] Chang TK, Ho P, Liang CT, Yu CK. Effects of vaginal septa on the reproductive performance of BALB/cByJNarl mice. J Am Assoc Lab Anim Sci. 2013; 52:520–3. 24041204 PMC3784654

[30] Bernardo V, Lourenço SQ, Cruz R, Monteiro-Leal LH, Silva LE, Camisasca DR, Farina M, Lins U. Reproducibility of immunostaining quantification and description of a new digital image processing procedure for quantitative evaluation of immunohistochemistry in pathology. Microsc Microanal. 2009; 15:353–65. 10.1017/S1431927609090710 19575836

[31] Leise MD, Poterucha JJ, Talwalkar JA. Drug-induced liver injury. Mayo Clin Proc. 2014; 89:95–106. 10.1016/j.mayocp.2013.09.016 24388027

[32] Shen T, Liu Y, Shang J, Xie Q, Li J, Yan M, Xu J, Niu J, Liu J, Watkins PB, Aithal GP, Andrade RJ, Dou X, et al. Incidence and Etiology of Drug-Induced Liver Injury in Mainland China. Gastroenterology. 2019; 156:2230–41.e11. 10.1053/j.gastro.2019.02.002 30742832

[33] Stine JG, Lewis JH. Current and future directions in the treatment and prevention of drug-induced liver injury: a systematic review. Expert Rev Gastroenterol Hepatol. 2016; 10:517–36. 10.1586/17474124.2016.1127756 26633044 PMC5074808

[34] Björnsson ES. Epidemiology, Predisposing Factors, and Outcomes of Drug-Induced Liver Injury. Clin Liver Dis. 2020; 24:1–10. 10.1016/j.cld.2019.08.002 31753242

[35] Louvet A, Ntandja Wandji LC, Lemaître E, Khaldi M, Lafforgue C, Artru F, Quesnel B, Lassailly G, Dharancy S, Mathurin P. Acute Liver Injury With Therapeutic Doses of Acetaminophen: A Prospective Study. Hepatology. 2021; 73:1945–55. 10.1002/hep.31678 33306215

[36] Hendrickson RG. What is the most appropriate dose of *N*-acetylcysteine after massive acetaminophen overdose? Clin Toxicol (Phila). 2019; 57:686–91. 10.1080/15563650.2019.1579914 30777470

[37] Cheng Y, Xie Y, Ge JC, Wang L, Peng DY, Yu NJ, Zhang Y, Jiang YH, Luo JP, Chen WD. Structural characterization and hepatoprotective activity of a galactoglucan from Poria cocos. Carbohydr Polym. 2021; 263:117979. 10.1016/j.carbpol.2021.117979 33858575

[38] Wu A, Feng B, Yu J, Yan L, Che L, Zhuo Y, Luo Y, Yu B, Wu D, Chen D. Fibroblast growth factor 21 attenuates iron overload-induced liver injury and fibrosis by inhibiting ferroptosis. Redox Biol. 2021; 46:102131. 10.1016/j.redox.2021.102131 34530349 PMC8445902

[39] Yang X, Jin Z, Lin D, Shen T, Zhang J, Li D, Wang X, Zhang C, Lin Z, Li X, Gong F. FGF21 alleviates acute liver injury by inducing the SIRT1-autophagy signalling pathway. J Cell Mol Med. 2022; 26:868–79. 10.1111/jcmm.17144 34984826 PMC8817117

[40] Hu X, Ni J, Gao N, Ye Z, Hu G, Cai J, Qian J. The effect of CYP3A4 genetic polymorphism and drug interaction on the metabolism of istradefylline. Chem Biol Interact. 2022; 366:110123. 10.1016/j.cbi.2022.110123 36007633

[41] Santoh M, Sanoh S, Takagi M, Ejiri Y, Kotake Y, Ohta S. Acetaminophen induces accumulation of functional rat CYP3A via polyubiquitination dysfunction. Sci Rep. 2016; 6:21373. 10.1038/srep21373 26900149 PMC4761967

[42] Dong HY, Shao JW, Wang T, Guo YH, Yan LY. [Effects on the activities and mRNA expression of CYP3A in rat's liver by four kinds of extracts from anti-cancer Traditional Chinese Medicines]. Zhong Yao Cai. 2008; 31:68–71. 18589752

[43] Pautz A, Li H, Kleinert H. Regulation of NOS expression in vascular diseases. Front Biosci (Landmark Ed). 2021; 26:85–101. 10.52586/4926 34027652

[44] Hsu CY, Lin YC, Chang LY, Huang SK, Huang CH, Yang CK, Huang CT, Lin CY. Therapeutic Role of Inducible Nitric Oxide Synthase Expressing Myeloid-Derived Suppressor Cells in Acetaminophen-Induced Murine Liver Failure. Front Immunol. 2020; 11:574839. 10.3389/fimmu.2020.574839 33250891 PMC7673381

[45] Lee KY, You HJ, Jeong HG, Kang JS, Kim HM, Rhee SD, Jeon YJ. Polysaccharide isolated from Poria cocos sclerotium induces NF-kappaB/Rel activation and iNOS expression through the activation of p38 kinase in murine macrophages. Int Immunopharmacol. 2004; 4:1029–38. 10.1016/j.intimp.2004.03.014 15222977

[46] Costa G, Pollack AE. Prenatal and postnatal drug exposure: focus on persistent central effects. Neural Regen Res. 2023; 18:1697–702. 10.4103/1673-5374.363190 36751782 PMC10154500

